# Submucosal gland neoplasms of the esophagus: an update and review

**DOI:** 10.1007/s10388-020-00758-1

**Published:** 2020-07-03

**Authors:** Ling Nie, Wei Li, Liyan Xue, Lin Wang, Yonghua Shen, Xiangshan Fan

**Affiliations:** 1 Department of Pathology, The Affiliated Drum Tower Hospital, Nanjing University Medical School, Jiangsu Province 210008 Nanjing, People’s Republic of China; 2 Department of Pathology, Cancer Institute and Cancer Hospital, Chinese Academy of Medical Sciences and Peking Union Medical College, 100021 Beijing, People’s Republic of China; 3 Department of Pathology, Shanxi Bethune Hospital, Shanxi Province 030032 Taiyuan, People’s Republic of China; 4 Department of Gastroenterology, The Affiliated Drum Tower Hospital, Nanjing University Medical School, Jiangsu Province 210008 Nanjing, People’s Republic of China

**Keywords:** Esophagus, Submucosal glands, Tumor, Classification

## Abstract

Submucosal glands (SMGs) present throughout human esophagus with clusters at either the upper third or lower third of the organ. SMGs tend to atrophy with age, and neoplasms arising in these glands are rare. In order to bring convenience to diagnosis, we summarize the histopathologic characteristics of all esophageal submucosal gland tumors (SGTs). Due to the morphological similarity, the nomenclature of salivary tumors is adopted for SGTs. However, there is great confusion about the definition and histogenesis of these tumors, especially the malignant subtypes. In the literature, esophageal mucoepidermoid carcinoma and adenoid cystic carcinoma usually adjoin the surface squamous epithelium and coexist with intraepithelial neoplasia or invasive squamous cell carcinoma (SCC). In addition, the typical gene alterations of salivary tumors have not been reported in these SGTs. Therefore, we propose to apply stringent diagnostic criteria to esophageal SGTs so as to exclude mimickers that are SCCs with various degree of SMG differentiation.

## Introduction

Esophageal submucosal gland (SMG) is located underneath the esophageal muscularis mucosa. It consists of mucous cells with or without a minor serous component and produces acid mucins and bicarbonate [1]. The duct penetrates through the mucosa to open into the esophageal lumen. Esophageal neoplasms arising in the SMG are rare. Due to the morphological similarity, the nomenclature of salivary tumors is adopted. The main types are mucoepidermoid carcinoma and adenoid cystic carcinoma [2, 3]. Over time, new entities have been discovered and characterized, and the spectrum of esophageal submucosal gland tumors (SGTs) has been expanded. However, most reported SGTs differ from salivary gland primaries. Awareness of the discrepancy is necessary to correct diagnosis and patient management.

This review summarizes reported esophageal SGTs (Table 1) with particular emphasis on controversies with regard to histology and classification. Attention is also paid to immunohistochemical markers and molecular alterations that can aid in the diagnostic work up of these neoplasms.

**Table 1:** The classification of esophageal submucosal gland neoplasms

Histological types
Benign
Submucosal gland duct adenoma
Canalicular Adenoma
Basal cell adenoma*
Oncocytoma*
Malignant
Secretory carcinoma
Acinic cell carcinoma
Adenoid cystic carcinoma
Mucoepidermoid carcinoma
Adenocarcinoma*

The asterisk (*) represents uncertain subtypes

## Benign SGTs

### Submucosal gland duct adenoma

Esophageal submucosal gland duct adenoma (SGDA) generally occurs in elderly patients with male predominance. The main symptom is abdominal discomfort and dysphagia. They are small hemispherical submucosal lesion that could be removed by endoscopic submucosal dissection (ESD) or endoscopic mucosal resection (EMR) [4]. Although two cases in the literature coexist with carcinoma [5, 6], the prognosis of esophageal SGDA itself is favorable.

**Fig. 1:**
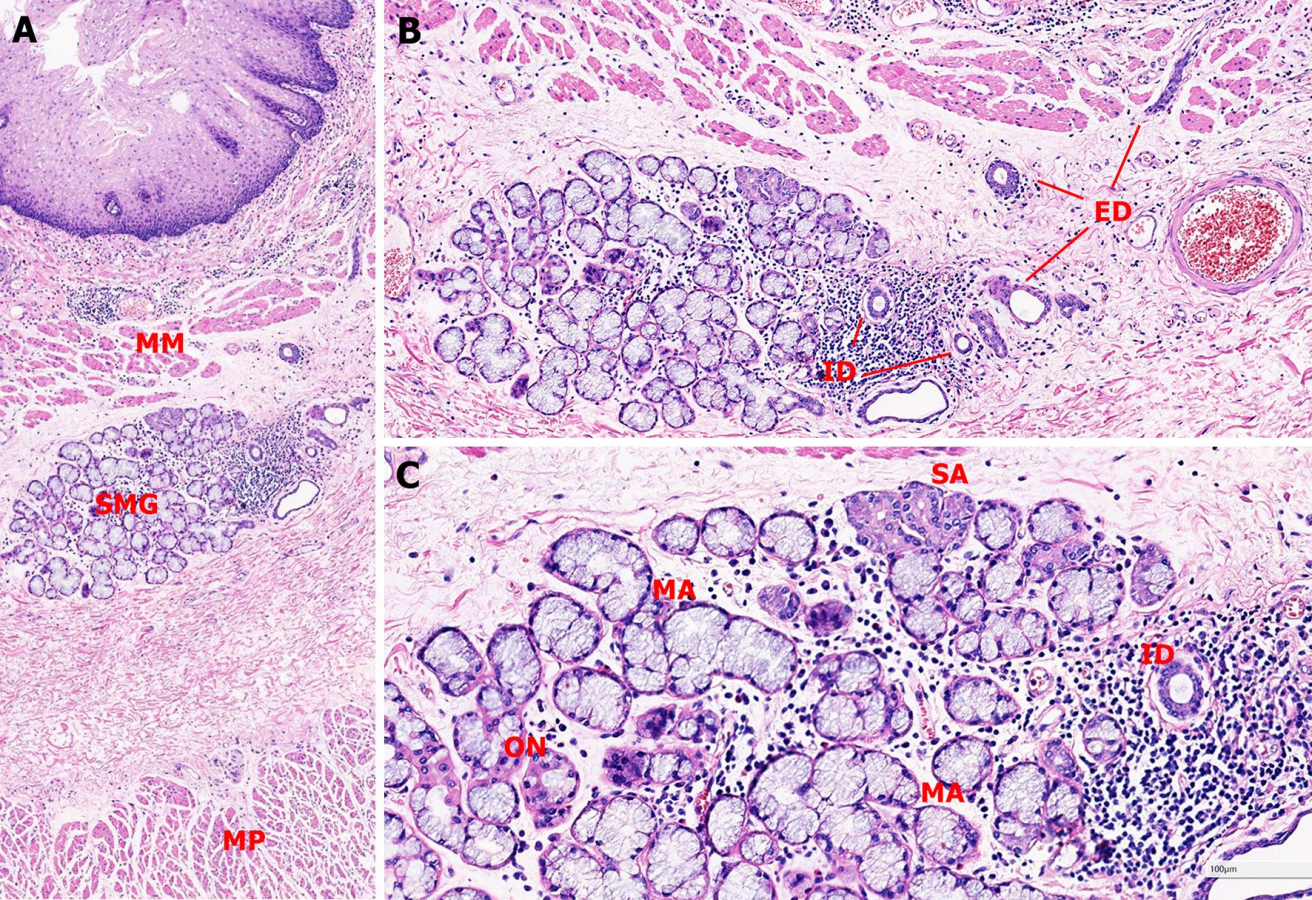
Normal structure of esophageal SMG. **a** The esophageal SMG locates at submucosa layer that between muscularis mucosa (MM) and muscularis propria (MP), the duct penetrates through MM to open into the esophageal lumen (25 ×). **b** The duct within the lobular area is called intralobular duct (ID), while the distal part is extralobular duct (ED) (50 ×). **c** SMGs are almost composed of pure mucous acini (MA), however, serous acini (SA) can be observed occasionally. Oncocytes (ON) are more common than serous cells (100 ×)

**Fig. 2:**
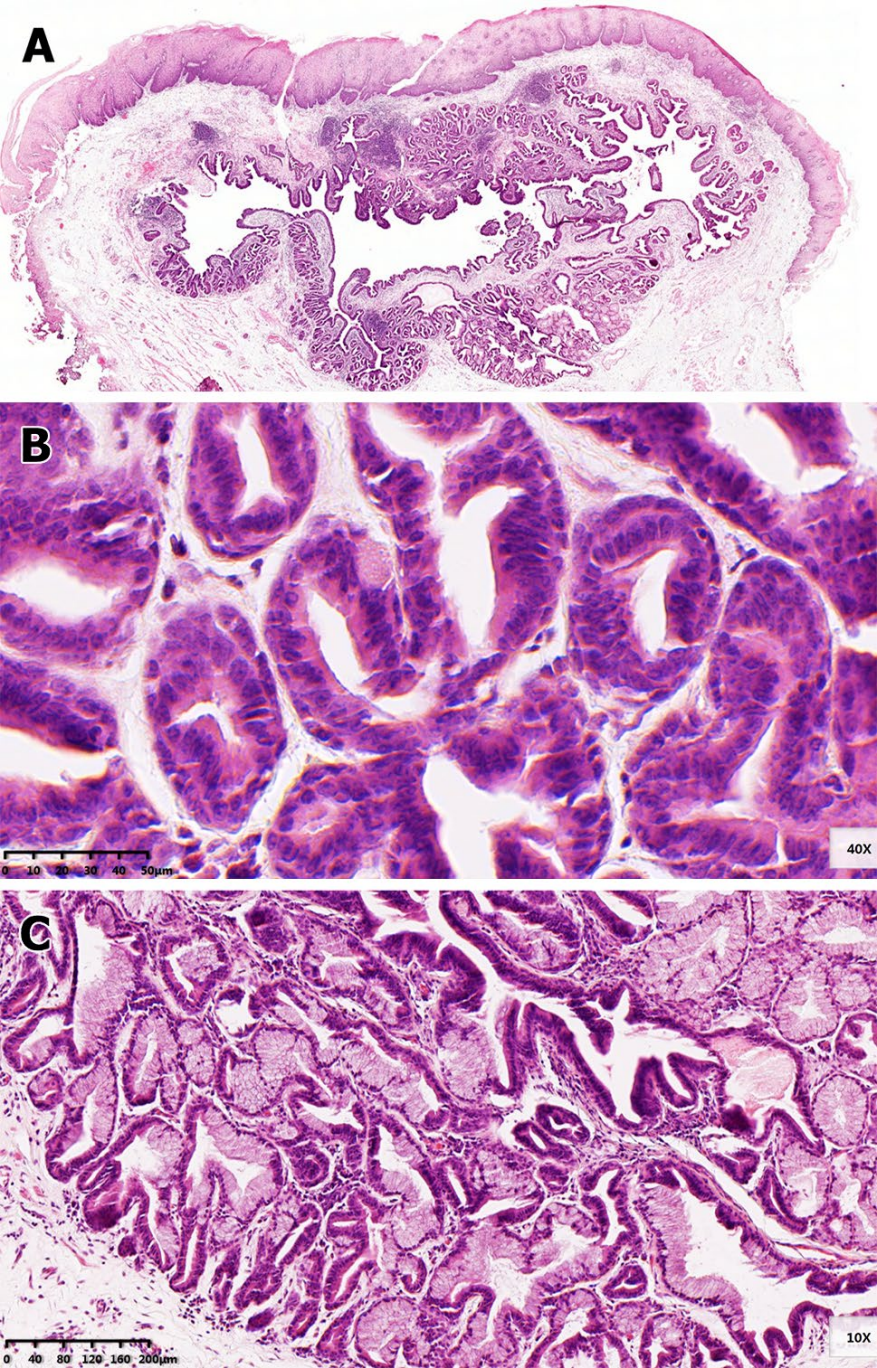
Histological features of esophageal submucosal gland duct adenoma (SGDA). **a** Under low magnification, the esophageal SGDA is located at submucosa presenting lobular structure with central cyst and focal lymphocytic aggregation. **b** It consists of multiple glands that covered by two layers of cells. The hyperplastic cells do not show significant atypia (400 ×). **c** Conspicuous ductal metaplasia in the concomitant acini (100 ×)

The SMG duct can be divided into intralobular and extralobular section (Fig. 1). The intralobular duct is lined by two-layered cuboidal epithelial and myoepithelial cells, while the extralobular duct is lined by two-layered columnar epithelial and basal cells, becoming squamous cells at the opening [1]. Most SGDAs display extralobular duct differentiation with stratified columnar epithelial and basal cells [7], while a few SGDAs show intralobular duct differentiation with stratified cuboidal epithelial and myoepithelial cells [5]. All these different cells could be detected in one case [8]. In rare circumstance, the surface of SGDA has a papillary structure covered by stratified squamous epithelium, reminiscent of sialadenoma papilliferum [9, 10]. According to the histological features, we proposed the diagnostic criteria of esophageal SGDA that include: (1) multiple glands or cysts that arranged as lobular structure and covered by two layers of cells, the inner luminal epithelial cells and the outer basal or myoepithelial cells; (2) presence of the multilayered epithelium and papillary structures in those glands or cysts, without marked cytologic atypia; (3) lymphocytic aggregation, and atrophy or disappearance of the concomitant acini (Fig. 2) [4]. The luminal lining cells are positive for MUC5B and various cytokeratins (CKs), including CK5/6, CK7, CK17, CK18, CK19, and HMWCK, while the outer layer cells are positive for p63, and SMA in some cases. The proliferating index is low [4, 5]. Alcian blue (pH 2.5) and periodic acid Schiff (AB-PAS) staining of the esophageal SGDA highlight the basement membrane, and microvilli on the apical surface of the luminal duct cells, which is compatible with the findings of the MUC5B immunostaining [5].

Two cases of esophageal SGDA coexist with carcinoma, but the carcinoma is not a result of malignant transformation from SGDA. Either the histological features or immunohistochemical staining patterns of esophageal SGDA resemble that of normal SMG duct. We also find a progressive relationship between retention cyst and SGDA, the multilayered epithelium and papillary folds are prone to occur in retention cysts of large size [4]. Therefore, we consider extensive ductal metaplasia, hyperplasia and/or retention cyst formation is the basis or precursor lesion of esophageal SGDA. Molecular evidence and further evaluation are needed to support the neoplastic nature of esophageal SGDA.

### Canalicular adenoma

Grimm et al*.* reported a canalicular adenoma that occurs at the proximal esophagus (24 cm from the incisors) [11]. The tumor is small (7 mm) and consists of columnar epithelial cells forming thin, branching, and interconnected cords in a loose and vascular stroma. There is loose fibrous tissue surrounding it. The tumor shows a low mitotic activity without necrosis. Neoplastic cells are positive for CK, S100, vimentin. Both the histological features and immunohistochemical results are accordant with that of salivary canalicular adenoma, but the tumor is simultaneously positive for chromogranin and synaptophysin. The authors give an interpretation that the tumor displays biphasic differentiation.

### Basal cell adenoma

Pandey et al. reported a basal cell adenoma that presents as a protuberant mass in the distal esophagus (35 cm from the incisors) [12]. The tumor is 3 cm in size and consists of small glandular duct-like structure with double layer epithelium. There is hyaline solid material around the duct-like structures. The tumor is positive for epithelial and myoepithelial markers. The low Ki-67 index, clear boundary, and hyaline material suggests a diagnosis of basal cell adenoma, but other salivary-type tumors are not completely excluded.

### Others

Pleomorphic adenoma and oncocytoma of the esophagus have been reported in early literature [13, 14], but we cannot confirm the facticity because of the vague illustrations.

## Malignant SGTs

### Secretory carcinoma (SC)

Secretory carcinoma (SC), formerly named mammary analogue secretory carcinoma, is a low-grade carcinoma characterized by morphological resemblance to mammary secretory carcinoma and *ETV6-NTRK3* gene fusion [15]. Chang et al*.* reported a primary esophageal SC occurring in an 85-year old man [16]. The tumor is located in the proximal esophagus (22 cm from the incisor) with a size of 15 × 8 mm. Tumor cells have eosinophilic cytoplasm and regular vesicular nuclei. The representative cystic and papillary structures are detected. The diagnosis is further established by fluorescence in situ hybridization (FISH) analysis that shows the characteristic *ETV6-NTRK3* gene fusion.

### Acinic cell carcinoma (AcCC)

As we know, the parotid gland is of the serous type with rare mucous units. More than 90% AcCCs occur in the parotid glands, they commonly show serous acinus and intercalated duct differentiation, forming solid, microcystic, and follicular patterns. The most significant cytologic feature of AcCC is the basophilic granular cytoplasm that give a diastase-resistant positive PAS reaction. Both DOG1 and SOX10 are immunopositive [17].

**Fig. 3:**
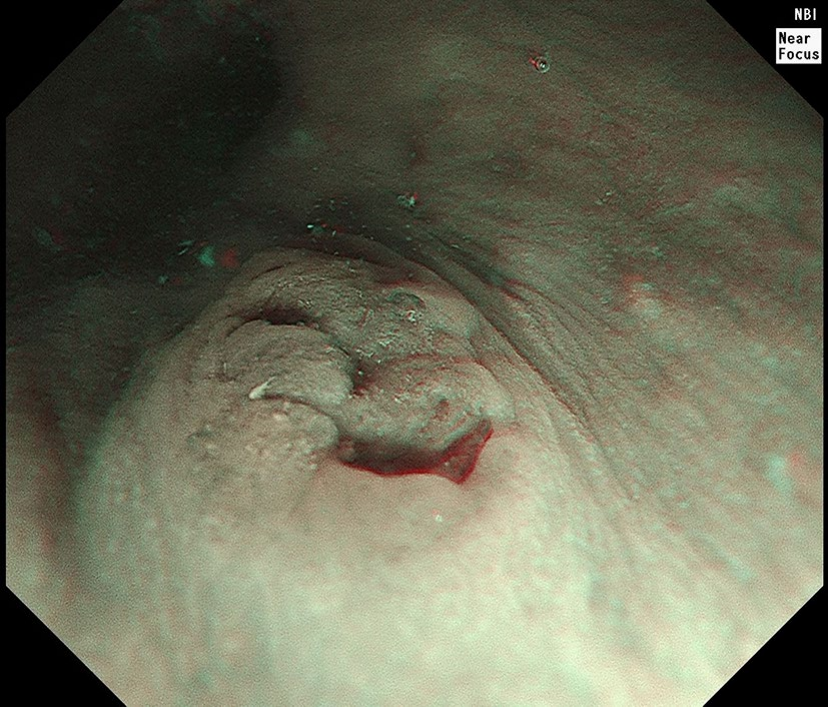
Narrow-band imaging of an esophageal acinic cell carcinoma (AcCC). Please note the rugged surface

**Fig. 4:**
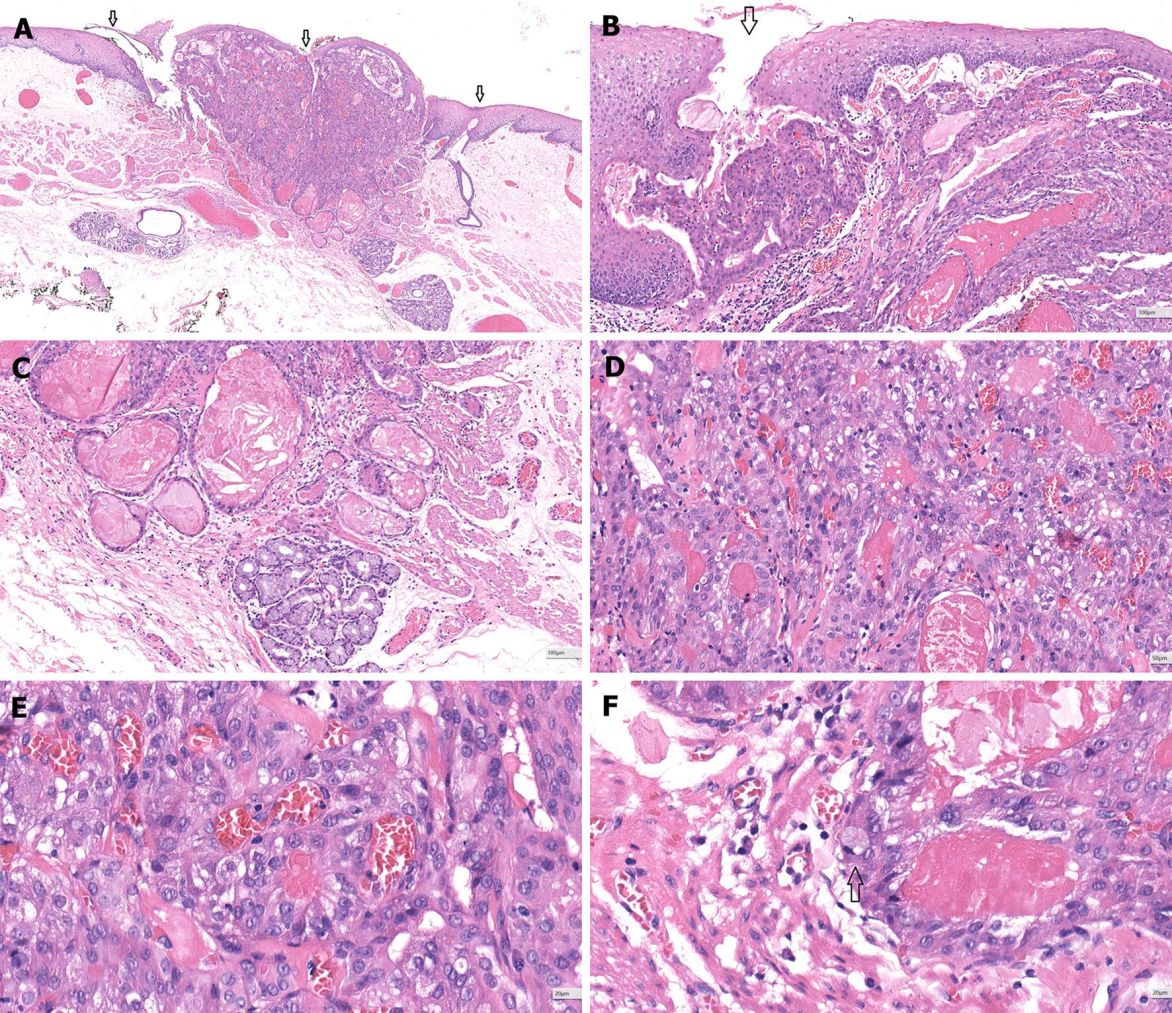
Histological features of esophageal AcCC. **a** The tumor is underneath the squamous epithelium and invade to focal submucosa (25 ×). The original extralobular ducts are destroyed by the tumor, but the ostia of these ducts are marked (arrows). **b** The tumor is covered by surface squamous epithelium without intraepithelial neoplasia (100 ×). Please note the ostium of SMG duct (arrows). **c** The minor ductal component of the tumor is adjacent to one SMG (100 ×). **d** Most area of the tumor shows the acinar differentiation (200 ×)**. e** The tumors cells have a uniform round or oval nucleus and eosinophilic or vacuolated cytoplasm (400 ×). **f** Mucous cell is rare but detectable (arrow) (400 ×)

**Fig. 5:**
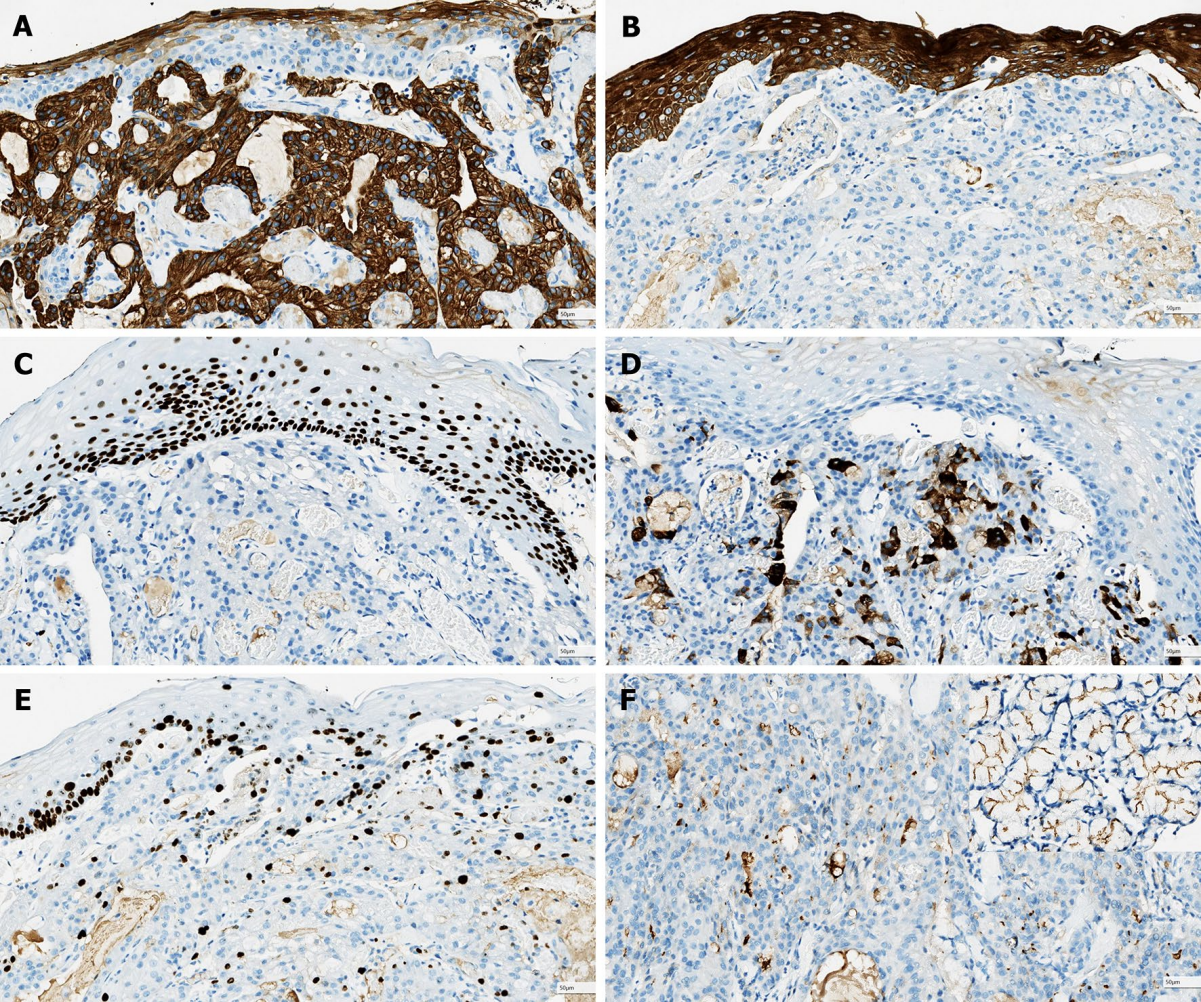
Immunohistochemical stain results of esophageal AcCC. **a** The tumor cells are positive for CK7. **b** CK5/6 is positive in the surface squamous cells but not in neoplastic cells. **C** The surface squamous cells are labeled by p63, but the tumor is negative staining. **d** MUC5AC labels the scattered mucous cells. **e** The proliferating rate indexed by K-i67 is low. **f** DOG1 is focal positive in the tumor. Please note the linear positivity of normal submucosal glands (inserted picture)

**Fig. 6:**
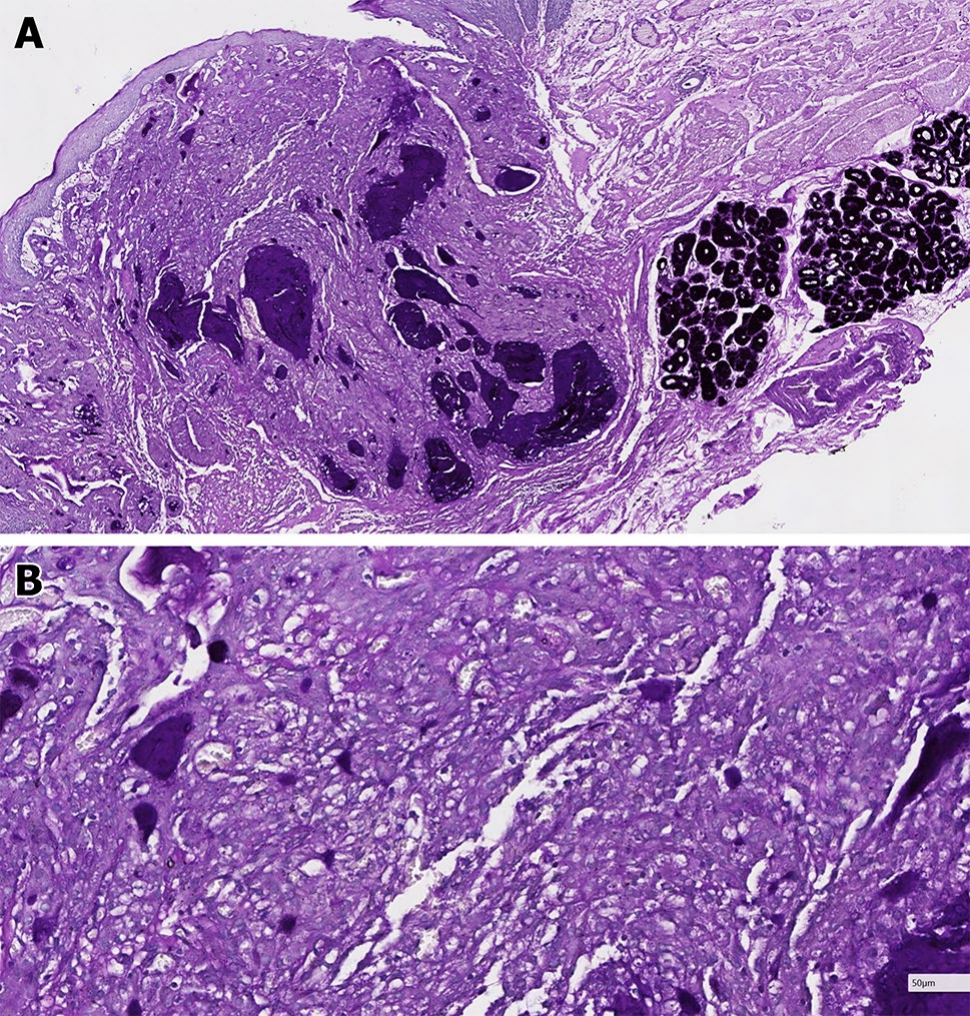
The diastase-resistant PAS stain result of esophageal AcCC. **a** The normal submucosal glands are strongly positive. **b** A few tumor cells demonstrate weak positivity

The two esophageal AcCC we diagnosed are both small lesion under endoscopy (Fig. 3). They are composed of major acinar structure, and minor ductal structure that filled with eosinophilic secretion. The nuclei of tumor cells are mild and uniform, and cytoplasm is eosinophilic or vacuolated (Fig. 4). The tumor cells are positive for CEA, CK7, and CK19 but negative for SOX10, TTF-1, p63, S100, Mammaglobin, and neuroendocrine markers. DOG1 is patchy positive or negative. Mucin markers may show scattered positivity, but mucous cells are rarely seen. The proliferating index is low (Fig. 5). The diastase-resistant PAS stain shows scattered weak positivity (Fig. 6) [18].

The esophageal SMGs are of mucous type with scarce serous cells [1]. In addition, the serous cells do not produce digestive zymogen but secrete solute and fluid with low concentration of proteins and peptides [19]. It is explainable that esophageal AcCCs show eosinophilic or vacuolated cytoplasm with scattered weak positivity of diastase-resistant PAS stain. Similar phenomenon has been observed in pulmonary AcCCs [20]. The origin of salivary AcCC has been traditionally sought among stem cells at the acinar-intercalated duct region [21, 22]. The diffuse immunohistochemical expression of DOG1 and SOX10 suggests that the histogenesis of AcCC simulates normal salivary embryogenetic events at the ends of branching rudiments [22–24]. Whereas, the expression of DOG1 and SOX10 in esophageal AcCC is unsatisfactory. This discordance implies that esophageal AcCC originates from extralobular duct.

We use the term AcCC to describe our two cases because of their resemblance to pulmonary AcCC, but considering the rarity and differences from salivary AcCC, it is still under debate. Another similar case is diagnosed as SGDA in the literature [25], but the monomorphism of neoplastic cells and absence of p63-positive basal and myoepithelial cells do not support the diagnosis. There is another lesion in the literature showing predominant mucous cell differentiation [26, 27], which is contrary to AcCC. A tumor lineage covering serous, mucous, and mixed type may exist in esophageal SGTs.

### Adenoid cystic carcinoma (AdCC)

Esophageal AdCC is defined as a malignant epithelial tumor of glandular differentiation, showing epithelial and myoepithelial cells in glandular or pseudoglandular lumina arranged in cribriform, tubular, or solid architecture [3]. The etiology and pathogenesis of esophageal AdCC remain unknown. However, over 80% traditional AdCCs have a definite activation of *MYB/MYB1* caused by gene fusion or other mechanisms [28]. No esophageal AdCC with the same gene alteration has been reported. Are they the same tumor type in different organs or just different tumors sharing similar morphology?

The name and definition of some tumors are changing with time. For example, the original term for AdCC is “cylindroma” based on the similar histological pattern [29]. When the concept of basaloid squamous cell carcinoma (BSCC) is newly proposed, the reported esophageal “AdCC” and “carcinoma with adenoid cystic differentiation” are more than twice of BSCC [30]. This is not the case in modern times, AdCC and BSCC constitute about 0.1% and 5% of all esophageal cancers, respectively [3, 31]. There are reviews focusing on esophageal AdCC at different periods in Japan [32, 33]. Comparing early and late cases, the clinical characteristics of AdCC in the former group are similar to those of squamous cell carcinoma (SCC), and most cases with an overlying squamous mucosa indeed have a small region of intraepithelial SCC [32]. While the latter group shows a lower ratio of lymph node metastasis and disease-related death, and an earlier tumor stage [33]. These differences are partly attributed to the consensus on SMG origin of AdCC, and the diagnosis of esophageal AdCC tends to be conservative [34]. Series report on BSCC demonstrates a histological diversity, including various proportion of solid nest, microcyst, trabecular nest, ductal differentiation, and cribriform pattern [35], but BSCCs with adenoid cystic features and those without are identical in many aspects [36]. Hence, most previously recognized esophageal AdCCs are gradually reclassified as BSCC.

**Fig. 7:**
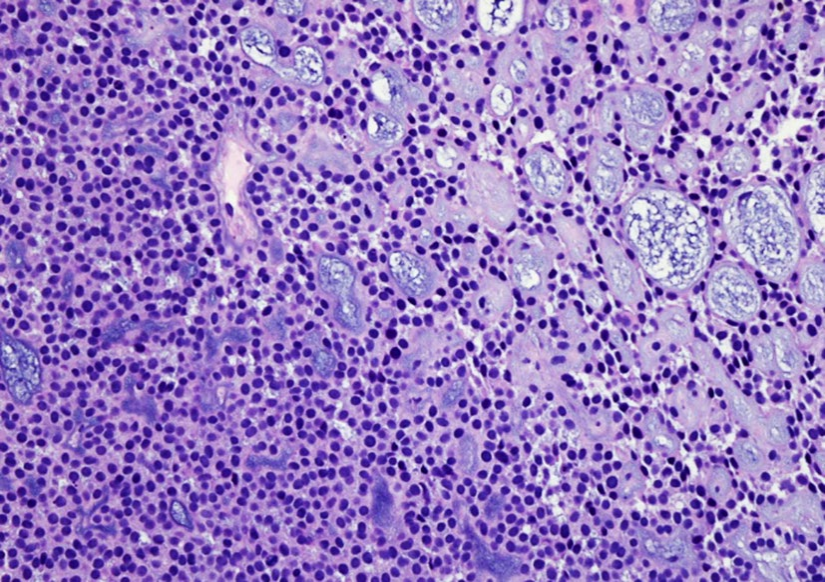
The esophageal adenoid cystic carcinoma (AdCC) is composed of impacted cells with scant cytoplasm, forming solid (left) and cribriform structures (right)

**Fig. 8:**
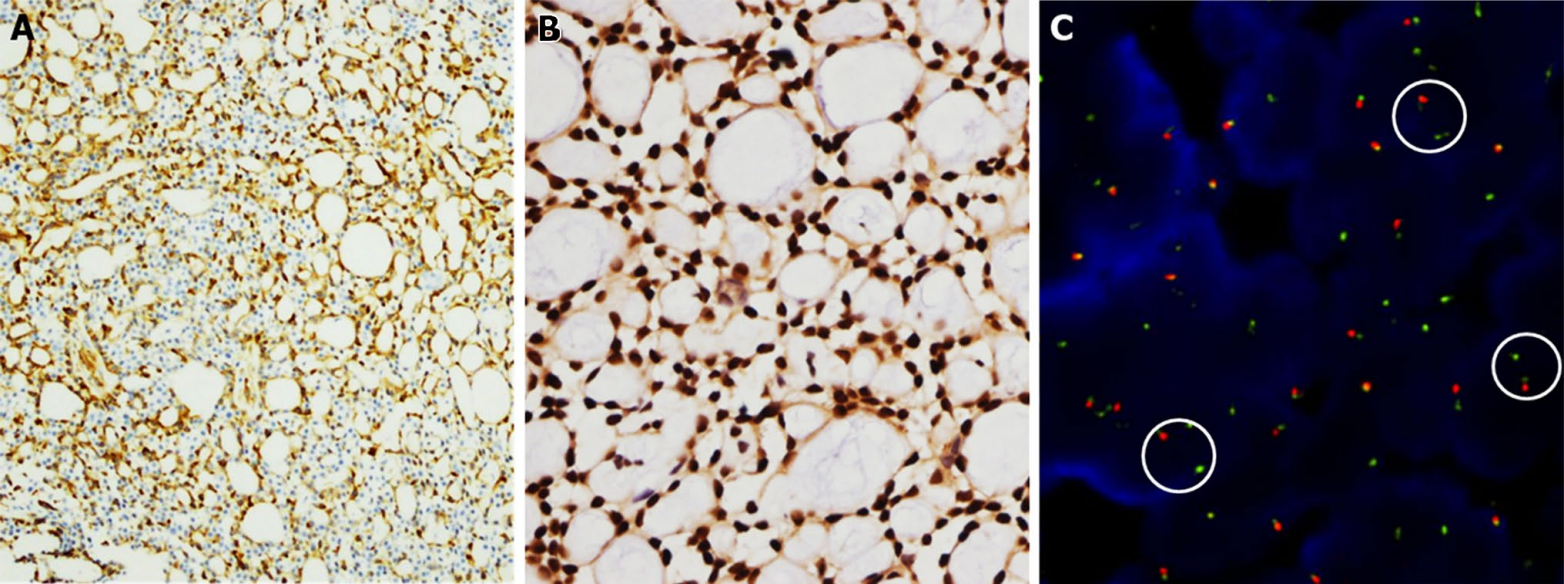
Immunohistochemical and FISH results of the esophageal AdCC. **a** The myoepithelial neoplastic cells are positive for Calponin (Calponin, CALP, zhongshanjinqiao, undiluted). **b** MYB (Anti-v-Myb + c-Myb (phospho S12), Abcam, 1:200) is diffusely positive. **c** FISH using a break-apart probe shows translocation of MYB gene (in the circle). The Zyto*Light* SPEC dual color break apart probe (PL100) is composed of ZyGreen (exciting 503 mm/ emission 528 mm) labeled polynucleotide, which target sequences mapping in 6q23.2–23.3 (chr6:134,840,690–135,483,752) proximal to the MYB breakpoint region. ZyOrange (exciting 547 mm/ emission 572 mm) labeled polynucleotide, which target sequences mapping in 6q23.3 (chr6:135,728,667–136,390,142) distal to the MYB breakpoint region

The esophageal SMG develops from the mucosal squamous epithelium at late phase (7th month) of gestation [34]. Theoretically, intraepithelial neoplasia, invasive SCC and BSCC all have the potential to differentiate toward SMG, forming tubular, microcystic, and cribriform structures. Sometimes, the SCC component is inconspicuous and desquamated during tumor progression, leaving a histological pattern of AdCC. If we accept the morphogenetic concept, these tumors are esophageal AdCC and accord with the criteria of WHO classification. However, the incidence rate of esophageal AdCC seems too high compared with the abovementioned SGTs. As we know, the salivary AdCC originates from the intercalated duct [37]. If we insist the histogenetic concept, only a few cases are true esophageal AdCCs, which have typical histological features and an intact surface squamous epithelium [38]. To date, we have encountered only one esophageal AdCC that is histologically, immunohistochemically, and genetically identical to salivary AdCC (Fig. 7, 8).

### Mucoepidermoid carcinoma (MEC)

Esophageal MEC is a neoplasm composed of an admixture of malignant epidermoid, intermediate, and mucous cells. The origin of esophageal MEC remains uncertain, but the best evidence suggests stem cells from the SMGs. This hypothesis has been widely accepted. It has also been suggested that MEC is simply SCC with diverging glandular differentiation [2]. Over one hundred esophageal MECs have been reported in the literature. Series report and comprehensive review of these tumors show the clinical characteristics are similar to that of esophageal SCC, while the patient outcome appears to have improved significantly during the past two decades [39, 40]. Most esophageal MECs show carcinoma in situ changes in the mucosa adjacent to the tumors [39]. The esophageal SCC frequently coexists with multiple primary carcinomas, glandular structure and mucus-secreting component [41, 42]. Considering the dispute on the origin and poor patient prognosis, the previously diagnosed esophageal MECs are probably a mixture of genuine MEC arising from SMG and SCC variant with SMG differentiation. Esophageal MEC with intact surface squamous epithelium is extremely rare [43].

Let us look back to the salivary gland MEC, it has become a clinically, morphologically and genetically heterogeneous entity. The positivity rate of gene translocation varies in different MEC variants [44]. However, no reported esophageal MEC has been demonstrated harboring the t (11;19) (q21;p13) translocation and CRTC1-MAML2 gene fusion or the t(11;15) (q21;q26) translocation and CRTC3-MAML2 gene fusion. We also fail to find a definite esophageal MEC in our database.

### Others

Although most esophageal adenocarcinomas arise from the Barrett’s mucosa, there are several cases of adenocarcinoma arising in SMG [45–50]. But some cases coexist with lesion of surface squamous epithelium, therefore the hypothesis of SMG origin is challenged [49, 50]. Some cases are more consilient to esophageal SGDA [46–48]. So, true esophageal submucosal gland duct adenocarcinoma is extremely rare [45]. Most of these cases possibly originate from squamous epithelium [51, 52]. At present, these esophageal cancers with significant SMG duct differentiation have not been comprehensively analyzed, and differential diagnosis relies on exclusive method.

## Conclusion

Although esophageal SGTs are rare, recognition of their distinct histopathologic characteristics is important to differential diagnosis. Most of these tumors have similar morphology to the salivary counterparts but differ in many aspects. Esophageal AcCC displays the unrepresentative histological features and immunohistochemical results. The reported esophageal AdCC and MEC are almost different entities to their salivary counterparts. They usually have high grade histological morphology, aggressive biological behavior and poor prognosis. In order to reduce the uncertainty and misunderstanding caused by using same nomenclature, we strongly suggest to apply stringent diagnostic criteria to esophageal SGTs, and presence of typical genetic alterations is necessary, especially for the esophageal AdCC and MEC. Moreover, a uniform term should be applied to describe the mimicker that is SCC with various degree of SMG differentiation. We also think grading other than elusive classification is significant to these mimickers. The squamoid and adenoid component can be graded separately. Many existing grading systems are applicable templates.
